# Stepwise plication of abdominal mesh technique for open abdomen closure

**DOI:** 10.1590/acb413426

**Published:** 2026-06-29

**Authors:** Diego Adão, Fauze Camargo Maluf, Gabriela Caetano Lopes Martins, Ramiro Colleoni, Milton Scalabrini, Leonardo De Mello Del Grande

**Affiliations:** 1 Universidade Federal de São Paulo – Escola Paulista de Medicina – Departamento de Cirurgia – São Paulo (SP) – Brazil.

**Keywords:** Abdominal Wound Closure Techniques, Open Abdomen Techniques, Surgical Mesh, Negative-Pressure Wound Therapy

## Abstract

**Purpose:**

To present the stepwise plication of abdominal mesh (SPAM) as a standardized dynamic closure technique that combines mesh-mediated fascial traction with negative pressure wound therapy for gradual abdominal wall approximation.

**Methods:**

In this technical report, we present SPAM for progressive closure of peritoneostomy with excessive abdominal wall retraction. It involves a central mesh incision followed by progressive tightening via hemostatic clamp rotation (similar to opening a can with a key), without full-thickness mesh cut. The mesh is secured with continuous suture and covered with a non-commercial negative pressure system. Cycles are repeated every 48–72 hours until fascial closure.

**Results:**

SPAM was applied in four patients: three successful cases (52-year-old male with rectal perforation, 36-year-old female post-Hartmann evisceration, 63-year-old female with gastrojejunal anastomosis leak) achieved primary fascial closure in a median of 10 days, with well-tolerated traction, no enteroatmospheric fistula, or incisional hernia. In one failure (59-year-old male with gastrojejunal leak post-laparoscopic gastrectomy), bleeding occurred during traction (controlled surgically), yet the technique was discontinued due to progressive clinical deterioration.

**Conclusion:**

SPAM offers a practical, standardized, reproducible approach to delayed fascial closure, particularly in resource-limited settings, enabling controlled progressive approximation without mesh resection.

## Introduction

The open abdomen is a temporary surgical strategy that plays a crucial role in emergency settings, particularly in cases of polytrauma and severe peritonitis, and has recently been associated with improved survival rates^1^. However, delaying the closure of an open abdomen is associated with significant morbidity and increased healthcare costs^2^. In the absence of an appropriate closure strategy, incisional hernia develops in 100% of patients, and enteroatmospheric fistula (EAF) formation has been reported in up to 8.9% of cases^3,4^. It contributed to an estimated financial burden of approximately USD 11,000 per patient^5^.

Various temporary abdominal coverage (TAC) and closure techniques have been described. In situations in which primary closure is not immediately feasible due to significant abdominal wall retraction, dynamic closure techniques combining mesh-mediated fascial traction with negative pressure wound therapy (MMFT–NPWT) seem the best choice^6,7^. Progressive mesh traction was initially described by Ferraz and Vieira^8^, and has been widely adopted since then, achieving closure success rates of up to 80%^9^. Petersson et al.^10^ first integrated MMFT with NPWT, originally suggesting that the mesh should be divided to facilitate tightening. Subsequent mesh traction techniques have exhibited considerable variability, but they consistently require mesh transection followed by suture fixation^11^. This increases both the duration and complexity of the procedure and does not allow the surgeon to adequately feel the degree of traction applied to the abdominal wall, which may end up being excessive or insufficient, depending on the amount of mesh that is cut.

This study proposed a standardized approach to progressive mesh traction by introducing a MMFT–NPWT technical modification, the stepwise plication of abdominal mesh (SPAM). We designed it to facilitate gradual and controlled abdominal wall approximation without mesh division in patients requiring delayed fascial closure.

## Methods

This technical report presents a comprehensive description of the SPAM technique, which was developed and implemented at the quaternary university hospital of Universidade Federal de São Paulo, Brazil. The procedures were conducted in sterile operating rooms and bedsides in 2024 and 2025. The reporting of the surgical technique was guided by adherence to the Surgical Technique Reporting Checklist and Standards (SUPER)^12^. Before the procedure, patients received detailed instructions, and informed consent was obtained. The study received approval from the institutional ethics committee Institutional Review Board—approval number 7,985,990 (CAAE 90411825.0.0000.5505)—, on November 18, 2025.

### Patient selection

The SPAM technique is indicated for adult patients (> 16 years old, any sex) requiring progressive closure of an open abdomen, particularly in cases in which primary fascial closure is not immediately feasible due to excessive lateral retraction of the abdominal wall. This technique is particularly useful in patients undergoing damage control surgery, severe intra-abdominal sepsis, or trauma-related peritoneostomy. SPAM should not be performed in patients with hemodynamic instability, complete fascial loss, uncontrolled intra-abdominal sepsis, enteric fistulas, and abdominal compartment syndrome.

### Materials

The materials required for the SPAM technique include a minor surgery tray, a 60 × 60 cm sterile plastic sheet, a 30 × 30 cm polypropylene mesh, 0 or 2-0 polypropylene suture (five units), gauzes (two or four units), a 16 Fr nasogastric catheter, and a sterile adhesive wound dressing. The estimated cost of these materials at our institution was approximately USD 45.29.

Surgeons needed to wear personal protective equipment, including masks, caps, and sterile gloves. After preparing the necessary materials, the procedure was performed in five steps. Prior to surgery, all patients received preoperative antibiotic prophylaxis.

### Step 1: Open abdomen preparation (in the surgical theater)

All patients underwent open abdomen management using a non-commercial negative pressure therapy system referred to at our institution as trans-abdominal anterior vacuum-assisted closure (TAbAVAC). The TAbAVAC is performed during the first revision surgery of the open abdomen, usually 48 hours after the damage control surgery, once the patient is clinically stable. In this procedure, when primary closure is not feasible, TAbAVAC is implemented as a preparatory step for the SPAM technique, which will be performed within the next 48 hours.

**Figure 1:**
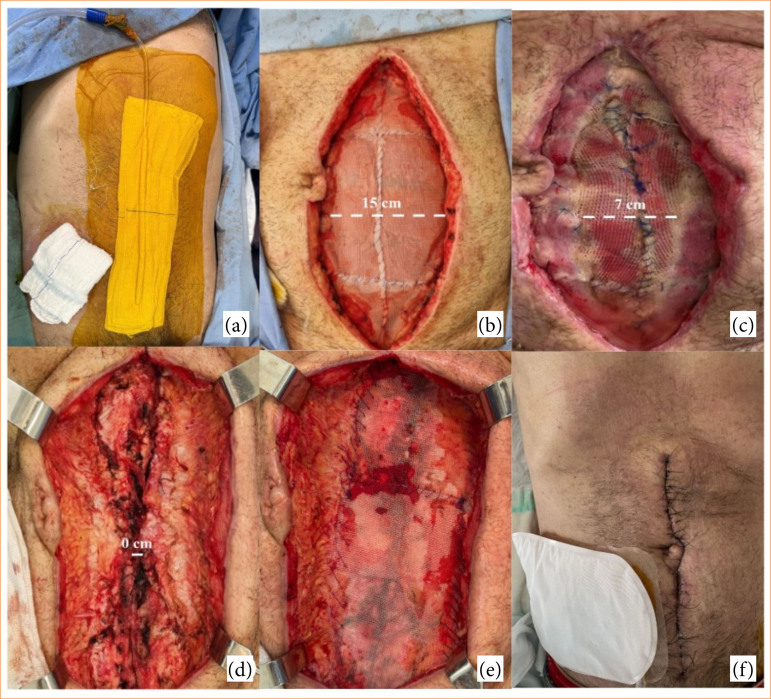
Stepwise plication of abdominal mesh (SPAM) technique phases. (a) Preparation of the open abdomen using the trans-abdominal anterior vacuum-assisted closure system. (b) At the initial operation, a maximum aponeurotic separation of 15 cm is observed, (c and d) followed by progressive approximation. Similar progression was observed in the first three cases reported. Complete tension-free primary aponeurotic closure was achieved after 14 days, followed by (e) onlay mesh placement and (f) definitive skin closure.

This system (a low-cost variation of the technique described by Barker) comprises a sterile, one-layer multi-perforated plastic sac to protect the intestinal loops, a 30 cm × 30 cm polypropylene mesh secured to the aponeurosis with a 3-cm overlap using continuous suturing in the quadrants (with minimal traction to avoid aponeurotic tearing), and a negative pressure dressing applied over the mesh^13^. One or two layers of gauze are placed between the abdominal wound edges, with a 16 Fr nasogastric catheter positioned within the gauze to facilitate drainage and maintain continuous suction (-60 to -90 mm Hg). The entire system is sealed with a sterile adhesive wound dressing (Fig. 1a).

After 48 hours of TAbAVAC, as the mesh begins to adhere to the aponeurosis, traction can be initiated at the bedside.

### Step 2: Patient positioning and sedation (at the bedside)

Patients were positioned in a supine or semi-Fowler position, depending on the surgeon’s preference, with continuous cardiovascular monitoring and supplemental oxygen administered via nasal cannula. Sedation and analgesia were achieved with intravenous ketamine (0.1 mg/kg) combined with midazolam (0.1 mg/kg), ensuring patient comfort while maintaining spontaneous respiration. Surgical drapes were positioned around the patient’s abdomen to ensure organization and maintain a clean field. The sterile adhesive dressing and gauze pads (TAbAVAC) were then removed, exposing the mesh and preparing it for the initiation of the SPAM technique.

### Step 3: Mesh traction

The SPAM technique began with a small longitudinal or transverse incision (~0.5 cm) in the central portion of the polypropylene mesh. A long, straight hemostatic clamp (e.g., Kocher) was inserted through this opening and rotated around its axis (clockwise or counterclockwise depending on the mesh symmetry, and ranging from 180 to 360°), progressively tightening the mesh and approximating the aponeurosis. This maneuver mimicked the mechanical effect of opening a key can, gradually increasing abdominal wall tension (video part 1, see Supplementary files)^14^. The number of rotations should be sufficient to generate traction but not excessive, to avoid compromising mesh fixation, tearing the aponeurosis, or causing a significant increase in intra-abdominal pressure.

### Step 4: Continuous suture fixation

After the traction rotation, a continuous suture was performed using 0 or 2-0 polypropylene along edges of the ridge created by the clamp’s long-axis rotation, beginning from the distal end to the base of the clamp (video part 2, see Supplementary files)^14^. The clamp was progressively withdrawn as the suture advanced, ensuring controlled mesh traction (at this point, additional rotations could be performed to increase or decrease the traction force along the midline, allowing for individualized adjustment throughout the abdominal wall). When passing the needle at the base of the mesh, caution was required to keep the suture superficial, avoiding the fixation of an intestinal loop beneath the sterile sac. In cases in which there was a high risk of enteropexy, Greek bar suture was advised.

After traction, new gauze pads and a sterile adhesive dressing were applied, reestablishing the TAbAVAC system. It is recommended to repeat mesh traction (steps 2 to 4) every 48–72 hours until complete fascial closure is achieved (Figs. 1b–1d).

### Step 5: Postoperative considerations

During the period of continuous traction, some patients may experience mild pain, and analgesia should be administered as needed. Caution is necessary, as excessive traction can cause severe pain, bleeding due to aponeurotic laceration, and an increase in intra-abdominal pressure. Measurement of intra-abdominal pressure is recommended after the first SPAM session. If the measured pressure is below 12 mm Hg, we do not routinely measure intra-abdominal pressure during subsequent sessions, as traction is always applied judiciously. Monitoring is recommended if excessive traction is suspected.

One or two planned returns to the operating room may be performed for gauze exchange and reinforcement of mesh fixation to the aponeurosis. In most cases, complete approximation is achieved within seven to 10 days. It is essential not to exceed 10 to 14 days, as mesh integration with the aponeurosis begins to occur, making its removal more difficult. Additionally, the intestinal loops start to form significant adhesions with each other and the parietal peritoneum, complicating midline approximation and closure.

During the final surgery, the dressing, the old mesh, and the plastic sheet should be removed, followed by primary closure using the small-bites technique. To reinforce the closure and minimize the risk of postoperative herniation, a new prophylactic onlay mesh should be placed. The skin is closed with sutures or staples, and a subcutaneous drain is optional, depending on the protocol of each institution (Figs. 1e and 1f).

## Results

The SPAM technique was performed in four patients at our institution.

### Case 1

A 52-year-old male patient with no relevant comorbidities presented with an open abdomen following emergency surgery for a perforated hollow viscus secondary to a rectal impalement injury. He developed septic shock due to severe peritonitis (ASA 1, PIPAS 4, Björk 1B), with intestinal loop edema and an inability to achieve primary closure. Initial management included primary repair of the rectal lesion, TAbAVAC, and clinical stabilization in the intensive care unit.

After 48 hours of clinical stabilization, the SPAM technique was initiated at the bedside to facilitate progressive traction and approximation of the abdominal wall. During the first week, the patient experienced two episodes of bleeding at the dressing site, aggravated by anticoagulation due to a thrombotic event, requiring surgical re-exploration for hemostasis revision. Although these events did not necessitate the interruption of mesh traction, they resulted in a four-day delay. Complete fascial closure was achieved after 13 days, allowing for primary closure using the small-bites technique with prophylactic onlay mesh. The patient had no complications after discharge, with no evidence of EAF or incisional hernia on follow-up.

### Case 2

A 36-year-old female patient with Castleman disease, who had been critically ill and hospitalized in the intensive care unit for a prolonged period due to acute cholangitis caused by a multidrug-resistant pathogen, developed an open abdomen after evisceration following a Hartmann’s procedure for an iatrogenic sigmoid perforation after colonoscopy. She had severe malnutrition and ongoing infection (ASA 3, PIPAS 3, Björk 2A). After clinical stabilization, on the fifth day of Barker vacuum therapy due to evisceration, the patient underwent TAbAVAC, with the first SPAM session performed during the same surgery.

The SPAM technique achieved complete aponeurosis approximation with a total of five plication sessions performed over nine days. Pain levels were mild and effectively managed with non-opioid analgesics. The procedure was well tolerated, with no major complications. During follow-up, the patient remained free of complications, with no signs of EAF or incisional hernia.

### Case 3

A 63-year-old female patient with no significant comorbidities and a history of Fobi-Capella surgery 12 years ago developed gastrojejunal anastomotic obstruction due to migration of the gastric ring. Degastrectomy associated with removal of the ring was performed. On the fifth post-operative day, she developed septic shock due to gastrojejunal anastomosis leakage (ASA 1, PIPAS 4, Björk 2A), and required emergency surgery, which included omental patch repair of the fistula, gastrostomy of the excluded stomach, and Barker vacuum therapy. After seven days of negative pressure therapy and once clinical stabilization had been achieved, TAbAVAC was implemented.

Complete aponeurosis closure was obtained after four SPAM sessions performed over eight days, without adverse events. Throughout the follow-up period, the patient experienced no complications, and there were no findings suggestive of EAF or incisional hernia.

### Case 4

A 59-year-old male with locally advanced gastric adenocarcinoma underwent laparoscopic partial gastrectomy with Roux-en-Y gastrojejunostomy. On post-operative day 5, the patient developed a gastrojejunal anastomosis leak, leading to fecal peritonitis and septic shock (ASA 4E, PIPAS 3, Björk 2C). Emergency damage-control laparotomy was performed with anastomosis takedown, peritoneal lavage, and Barker vacuum therapy. The patient subsequently developed an EAF (ASA 4E, PIPAS 1, Björk 2C). Fistula management consisted of decompressive gastrostomy, proximal jejunostomy (biliopancreatic loop), distal jejunostomy for feeding, and Barker vacuum therapy. Subsequently, TAbAVAC was implemented and SPAM initiated. During mesh traction sessions, the patient evolved with local bleeding, requiring hemostatic and lavage procedure. In total, 14 lavage procedures were performed, and the patient presented several complications during follow-up, including bowel ischemia leading to extensive enterectomy and short bowel syndrome.

The patient continued to experience fascial retraction, and primary fascial closure was not achieved. SPAM was interrupted after three months, and exclusive palliative care was instituted due to progressive severe malnutrition. Abdominal skin closure was performed, and at the time of writing, the patient remained hospitalized under the care of the palliative care team.

## Discussion

We present four cases applying the SPAM technique, a variation of the classical MMFT–NPWT approach, in which the open abdomen is closed without the need for mesh resection, with tailored traction and protected intestinal loops. In this case series, three cases were successful, with no development of EAF or incisional hernia. In one case, fascial traction was discontinued due to the patient’s unfavorable clinical condition. SPAM was performed in a standardized fashion using simple, low-cost materials readily available in hospitals within low-income countries, facilitating its reproducibility and potential integration into a wide range of clinical environments.

Temporary peritoneostomy can be lifesaving in cases of abdominal compartment syndrome, severe peritonitis, or damage control surgery. However, prolonged maintenance of an open abdomen is associated with significant morbidity^15^. The absence of soft tissue hinders healing, and the exposure of bowels increases the likelihood of perforation, leading to a “vicious cycle” of EAF^16^. Pommerening et al.^17^ found that longer intervals before first re-operation was associated with a reduced probability of achieving primary fascial closure, with primary closure success rates declining by 1.1% for each hour beyond 24 hours following the initial surgery. Therefore, early closure strategies should be prioritized to reduce hospital stay and mortality rates^15^.

Early fascial closure mainly relies on adequate intensive care management, whereas delayed closure depends on TAC technique^18^. Several progressive closure strategies have been described, including progressive fascial traction with retention sutures, commercially available vacuum-assisted closure (VAC) devices, and the Wittmann Patch method^19-21^. Ferraz and Vieira^8^ introduced a progressive closure technique for laparostomy using a dual temporary prosthesis of polypropylene mesh and polyamide sheet, applied to 23 patients with severe generalized suppurative peritonitis between 1990 and 1994 at the Hospital Universitário Clementino Fraga Filho (Universidade Federal do Rio de Janeiro), in Brazil. As for the introduction of negative pressure techniques, it gained prominence in 1995 following Barker et al.’s description of vacuum-filled dressings for temporarily sealing the abdominal cavity^22^. This approach later advanced into VAC therapy, as outlined by Garner et al.^23^, marking a significant evolution in the technique.

Later, Petersson et al.^10^ combined mesh-mediated fascial traction with VAC in seven patients between 2005 and 2006 at Malmö and Uppsala University Hospitals. However, while the authors described dividing the mesh, we proposed rolling it instead. Rotating the mesh (similar to turning a key in a can) rather than cutting it may prevent fascial retraction, and facilitate needle passage away from the intestinal loops due to protection provided by the clamp. Additionally, it allows for the calibration of traction and correction in cases of excessive force, allowing segment-by-segment adjustment of traction along the midline. In the previously proposed technique of cutting the mesh, incorrect or excessive trimming prevents traction adjustment, potentially resulting in excessive pressure or requiring mesh replacement. Future studies comparing both techniques are necessary.

There is no consensus in the literature about the use of dynamic closure techniques in open abdomen due to insufficient evidence, with only observational studies on MMFT–NPWT^24^. EHS clinical guidelines found pooled proportions of fascial closure to be higher with dynamic (75.9%) compared with static techniques (33.9%). Similarly, fistula formation is less frequent with dynamic closure (4.3%) than with static closure (11.9%). Mortality rates also favor dynamic techniques, at 16.3%, versus 25.5% with static methods^24^.

As for the integration of vacuum techniques, Petersson and Petersson11 conducted an updated systematic review of the MMFT–NPWT technique. Primary findings showed a weighted average fascial closure rate of 83.5%, achieved within seven to 32 days and requiring two to 10 dressing changes. The incidence of EAF was low (5.6%), and the planned ventral hernia rate was 6.2%. The WSES consensus paper found a weighted fascial closure rate of 53.9% with NPWT without MMFT, and 9.8% of EAF in prospective studies. In contrast, NPWT combined with fascial traction achieved a 77.8% closure rate and a 4.3% incidence of EAF^25^.

Existing guidelines recommendations on negative pressure values vary based on the technique employed and the approach to creating negative pressure. In the vacuum pack method outlined by Barker et al.^22^, a negative pressure range of 100–150 mm Hg is suggested to ensure an effective seal and improved handling of peritoneal fluid. For the VAC system (including the abdominal dressing system and ABThera), a negative pressure of 125 mm Hg is advised, as it is deemed essential for enhancing blood flow, promoting cellular proliferation, and optimizing tissue expansion, though this lacks support from clinical trials or prospective randomized studies. Certain authors advocate for reduced pressures (25–50 mm Hg) in scenarios with elevated bleeding risk or coagulopathy^23^.

Although effective, current dynamic closure techniques have limitations, such as high costs and the need for specialized infrastructure^26^. SPAM stands out as a low-cost and bedside-compatible approach that allows for precise tension control.

The safety profile of the technique is a critical factor, particularly the risk of ischemia and fascial necrosis due to inadequate traction. SPAM enables gradual and individual adjustments with simultaneous traction of entire suture line. Another major concern is the development of EAF, a severe complication observed in cases of prolonged open abdomen management, as exemplified by case 4 in our series^4^. The preservation of polypropylene mesh integrity, without the need for resection, may contribute to reducing intestinal exposure and, consequently, the risk of fistula formation, as the intestinal loops remain continuously protected by a plastic membrane. Additionally, the technique maintains continuous negative pressure therapy (TAbAVAC), which may reduce infection rates associated with progressive closure and potentially accelerate the time to definitive closure^27^.

The applicability of SPAM depends on careful patient selection. Ramírez-Giraldo et al.^28^ retrospectively evaluated 92 open abdomen cases managed with MMFT and identified a number of lavage procedures greater than four, intestinal resection and an initial fascial gap larger than 7.5 cm as the most important predictors of closure failure. In the fourth patient in our series, for whom fascial closure was not feasible, a total of 14 lavage procedures were performed, and the patient underwent intestinal resection, which may result from ischemia related to increased intra-abdominal pressure following abdominal closure. Therefore, the efficacy of SPAM, as well as other MMFT techniques, may be limited in patients who require multiple lavage procedures, undergo intestinal resection, or present with severe loss of domain. In case of excessive fascial separation, patients may require complementary strategies such as component separation techniques and the use of botulinum toxin^29^. These approaches, however, require surgeons with greater expertise in complex abdominal wall reconstruction and can increase both hospitalization costs and length of stay.

Three additional limitations of our technique are worth noting: the use of wall suction—whose pressure fluctuates throughout the day, failing to ensure a stable and predictable value—, the risk of bleeding, and the risk of abdominal compartment syndrome, which cannot be excluded as a contributing factor to the clinical deterioration observed in case 4. Nevertheless, since the vacuum is applied only to the superficial portion of the wound, it does not appear to increase the risk of developing an EAF. Regarding bleeding, this risk is inherent to any surgical procedure and was promptly controlled with surgery. As for the abdominal compartment syndrome, increased intra-abdominal pressure is a well-recognized risk associated with any abdominal closure technique. Although the SPAM technique aims to mitigate this risk, it cannot be completely eliminated. Therefore, patients undergoing this approach should be closely monitored for early signs of intra-abdominal hypertension.

Future research should focus on multicenter prospective studies to validate the clinical effectiveness of SPAM by assessing definitive closure rates, complications, costs, and the long-term impact on abdominal wall integrity. Additionally, direct comparisons between SPAM and other established techniques are essential to determine its relative efficacy, cost-effectiveness, and impact on patient quality of life.

## Conclusion

The prevention of complex incisional hernias secondary to an open abdomen remains a critical challenge in emergency and trauma surgery. The SPAM technique is a low-cost and bedside-compatible approach for open abdomen closure, providing gradual fascial approximation with controlled tension. It has the potential to reduce complications associated with excessive fascial traction and prolonged open abdomen maintenance. Additionally, by facilitating early closure, it may help prevent the development of complex incisional hernias. It offers a practical, standardized, and easily reproducible approach to delayed fascial closure, particularly in resource-limited settings. Further studies are needed to validate its long-term efficacy, impact on patient outcomes, and effectiveness compared to existing closure techniques.

## Data Availability

The data are available in a data repository: https://doi.org/10.5281/zenodo.20024563.
